# Anxiety in Multiple Sclerosis Patients: A Preliminary Examination of the Effect of Fatigue, Physical Disability, and Low Self-Compassionate Attitude

**DOI:** 10.1192/j.eurpsy.2022.495

**Published:** 2022-09-01

**Authors:** T. Carvalho, A.L. Côrte-Real Lopes Nunes, L. Benedito, C. Gomes

**Affiliations:** 1 Instituto Superior Miguel Torga (ISMT); Center for Research in Neuropsychology and Cognitive-Behavioral Intervention (CINEICC), University of Coimbra, Portugal, Psychology, Coimbra, Portugal; 2 Instituto Superior Miguel Torga, Escola De Altos Estudos, Coimbra, Portugal; 3 Instituto Superior Miguel Torga, Psychology, Coimbra, Portugal; 4 Clínica de Saúde Psiquiátrica de Coimbra (CSPC) – Casa da Oliveira, Psychology, Coimbra, Portugal

**Keywords:** multiple sclerosis, anxiety symptoms, predictive model

## Abstract

**Introduction:**

Multiple Sclerosis (MS) is a demyelinating, neurodegenerative, and immune-mediated disease that affects the central nervous system. Usually co-occurs with difficulties in emotional regulation and psychopathology. Anxiety is one of the most common psychiatric manifestations in patients with MS. Nonetheless, empirical evidences on the joint predictive effect of MS clinical conditions and emotion regulation processes on the development of anxiety in MS patients are scarce.

**Objectives:**

This preliminary study aimed to explore whether fatigue, physical disability (MS clinical conditions) and a low compassionate attitude (maladaptive emotion regulation process based on self-judgment, over-identification, and isolation) predict anxiety symptoms in MS patients.

**Methods:**

A convenience sample of 107 patients with MS diagnosis and without other neurological disorders was used in this cross-sectional study. Participants completed the Anxiety Subscale of the Depression, Anxiety and Stress Scales-21, the Analogic Fatigue Scale, the World Health Organization Disability Assessment Schedule, and the Self-judgment, Isolation and Over-identification Subscales of the Self-Compassion Scale.

**Results:**

All potential predictors showed significant correlations with anxiety symptoms and predicted this symptomatology through simple linear regressions. Therefore, they were selected as covariates of the multiple linear regression model, which explained 32% of the variance of anxiety symptoms. This model revealed that fatigue, physical disability, and low compassionate attitude are significant predictors.

**Conclusions:**

The results support the relevance of psychological interventions for MS patients to implement effective strategies to regulate anxiety associated with fatigue and physical disability. Helping patients to adopt a more compassionate attitude toward the self can reduce their anxiety.

**Disclosure:**

No significant relationships.

